# Emotional Dependency and Parasocial Grief Following the Alteration or Loss of Companion Artificial Intelligences: A Systematic Review

**DOI:** 10.3390/bs16091548

**Published:** 2026-09-01

**Authors:** Johanna Lilibeth Alcívar Ponce, Wilson Alexander Zambrano Vélez, Gioryi Sornoza Zavala, José Manuel Peñafiel Mejillones, Julio César Rivera Ruiz

**Affiliations:** Facultad de Ciencias Sociales y de la Salud, Universidad Estatal Península de Santa Elena, La Libertad 240250, Ecuador; jalcivar@upse.edu.ec (J.L.A.P.); gsornoza7749@upse.edu.ec (G.S.Z.); jose.penafielmejillones0073@upse.edu.ec (J.M.P.M.); jriverar2@unemi.edu.ec (J.C.R.R.)

**Keywords:** companion AI, parasocial grief, emotional dependency, technological disruption

## Abstract

With the advance of large language models, companion AI agents have evolved to simulate complex relational dynamics, responding to the need for affection and emotional support. This systematic review aimed to analyze the available empirical evidence on the manifestations of emotional dependency and parasocial grief experienced by users in response to the algorithmic alteration or loss of these companion AI agents. Following the guidelines of the PRISMA 2020 statement and with a protocol registered on OSF, an exhaustive search was conducted across the Web of Science, Scopus, PubMed, and APA PsycINFO databases. After applying inclusion criteria via the PICOS framework and evaluating methodological quality using the MMAT tool, 14 empirical studies were selected and included. The synthesized results suggest that technical disruptions (updates, content filters, or server shutdowns) are associated with disruptions in the agent’s identity continuity, which users frequently described as coinciding with separation distress, depressive symptoms, perceived isolation, and parasocial grief expressed through anthropomorphic metaphors. It is preliminarily concluded that the loss or modification of companion AI agents may be linked to a psychosocial impact that presents descriptive similarities to interpersonal breakups or disenfranchised grief.

## 1. Introduction

With the development of conversational technologies and increasing rates of social isolation (Fan et al., 2026), companion artificial intelligence (AI) agents, based on large language models (LLMs), have evolved from basic dialogue interfaces into agents designed to simulate responsive relational dynamics (Hasselberger & Lott, 2026; Nakagomi et al., 2026). Their adoption has been primarily driven by their potential to compensate for loneliness (Maples et al., 2024), leading users to assign them complex social roles ranging from therapeutic guidance support (Kouros & Papa, 2024) to the consolidation of friendship, kinship, or romantic companionship bonds on mass-consumer platforms (Djufril et al., 2025; Hasselberger & Lott, 2026).

However, although companion AI agents enable the establishment of deep emotional relationships by engaging the user’s sense of identity, risks such as emotional dependency also arise (Ma et al., 2026; Meng et al., 2023; Merrill et al., 2022). In interpersonal contexts, emotional dependency has been conceptualized as a pattern of excessive affective reliance that may involve compulsive behaviors and displacement of other support networks (Bution & Wechsler, 2016). In the context of companion AI agents, however, the available evidence does not establish this pattern as a clinical disorder. Such dynamics are primarily grounded in Attachment Theory, formulated by Bowlby (1969) and expanded by Ainsworth et al. (1978), which posits an innate human search for proximity to a figure that functions as a secure base and safe haven in the face of psychophysiological distress.

In this sense, the design of companion AI may engage relational processes associated with responsiveness, availability, perceived emotional support through the simulation of responsiveness, constant availability (Kasturiratna & Hartanto, 2026) and the structuring of a personalized relational memory (Siemon et al., 2026). By deploying strategies of simulated active listening and language of closeness, the technology positions itself before the user as a new safe haven (Maeda & Quan-Haase, 2024), promoting levels of deep self-disclosure (Li & Zhang, 2024), which may be associated with increased self-disclosure and, in some contexts, with changes in patterns of offline communication and interpersonal interaction (Yuan et al., 2024).

On the other hand, the adaptation of Horton and Wohl’s Parasocial Interaction Theory to the field of artificial intelligence reveals a substantial evolution regarding traditional digital entertainment media, replacing the passive reception of technological tools with personalized and bidirectional interactivity from companion AI agents (Horton & Wohl, 1956; Hung et al., 2026). In this environment, the perception of reciprocity fosters the establishment of asymmetrical yet profound affective bonds (Griffin & Powell, 2025). Consequently, the alteration or loss of the companion AI agent’s identity and memory triggers so-called parasocial grief, conceptualized as a legitimate response of psychological suffering in the face of relationship dissolution (DeGroot & Leith, 2018; Wullenkord & Brüggeshemke, 2025). Such emotional manifestation is equated to the distress generated by interpersonal breakups or losses in conventional fictional media, expressed through the use of death metaphors and clinical anguish responses (Banks, 2024; Kim et al., 2026; Poonsiriwong et al., 2026).

Disruptions in the continuity of interaction stem from specific technical triggers, such as updates in large language models, the abrupt implementation of safety filters, personality reconfigurations, or the definitive shutdown of servers (Banks, 2024; Nature Machine Intelligence, 2025). Unforeseen modifications of this nature cause severe service experience failures and prevent the achievement of cognitive closure, generating a feeling of ambiguous loss that freezes the grieving process (Kim et al., 2026; Poonsiriwong et al., 2026). Simultaneously, the absence of clear termination protocols may contribute to users’ psychological disorientation, increasing technological overdependency and leading to states of shock or digital abandonment (Richet, 2025; Soh et al., 2026; Zhang et al., 2024).

Various empirical investigations have associated continuous interaction with companion AI agents with indicators of emotional dependency and problematic patterns of use (Lin & Chien, 2024). In this regard, Richet (2025) reports the manifestation of digital entrapment, defined as a recurrent causal loop that alters the user’s relational expectations and reinforces the over-personification of the agent. This phenomenon is simultaneously sustained by mechanisms of cognitive dependency and affective involvement, as substantiated by Zhai et al. (2025) when analyzing functional motivations and psychic vulnerabilities. Likewise, recent evidence reveals that intense emotional bonding has been associated with psychological distress and adverse emotional reactions following disruption or loss of the AI relationship (Chen et al., 2025; Fang & Qiu, 2025).

Research documents a range of behavioral and emotional responses reported by users following the loss, alteration, or interruption of companion AI services. On the one hand, Zhang et al. (2024) identify escapism tactics and so-called “transferable withdrawal,” characterized by generalized avoidance and the rejection of interacting with any chatbot after a system failure. On the other hand, the literature records compensatory re-creation attempts aimed at restoring the interrupted bond. In such scenarios, users export conversation histories, capture personality traits, and design specific prompts to clone the digital companion on alternative platforms (Banks, 2024; Poonsiriwong et al., 2026).

Given the relevance of this relational dynamic, current empirical evidence remains fragmented, and the reviewed literature presents significant thematic gaps regarding the life cycles of human–AI bonds. Existing studies have primarily focused on the therapeutic utility of AI in addressing loneliness (Chou et al., 2023; Feng et al., 2025), functional programming parameters (Lim et al., 2026), or the application of technology to facilitate human grief processing (Soh et al., 2026). More recently, comprehensive syntheses such as that by Ho et al. (2025) have mapped the general benefits and risks of romantic companion AI using Sternberg’s Triangular Theory of Love, which conceptualizes emotional bonds within stable relational states. However, these contributions primarily evaluate the initial formation, customization, and static operational risks of companion AI agents, leaving a critical blind spot regarding the psychological consequences of involuntary disruption, service discontinuity, and relational dissolution.

This review differs significantly from and expands upon the work of Ho et al. (2025) by shifting the analytical focus from general attachment dynamics to specific psychosocial and behavioral phenomena following disruption. Therefore, the study centers on parasocial grief, ambiguous loss, and manifestations of emotional dependency triggered by abrupt model updates, security reconfigurations, or server closures while synthesizing user coping strategies and ethical termination protocols. By examining how involuntary system changes may disrupt users’ perceived sense of security and relational continuity, this review offers a novel framework that addresses a critical gap not covered in the previous literature.

**Figure 1:**
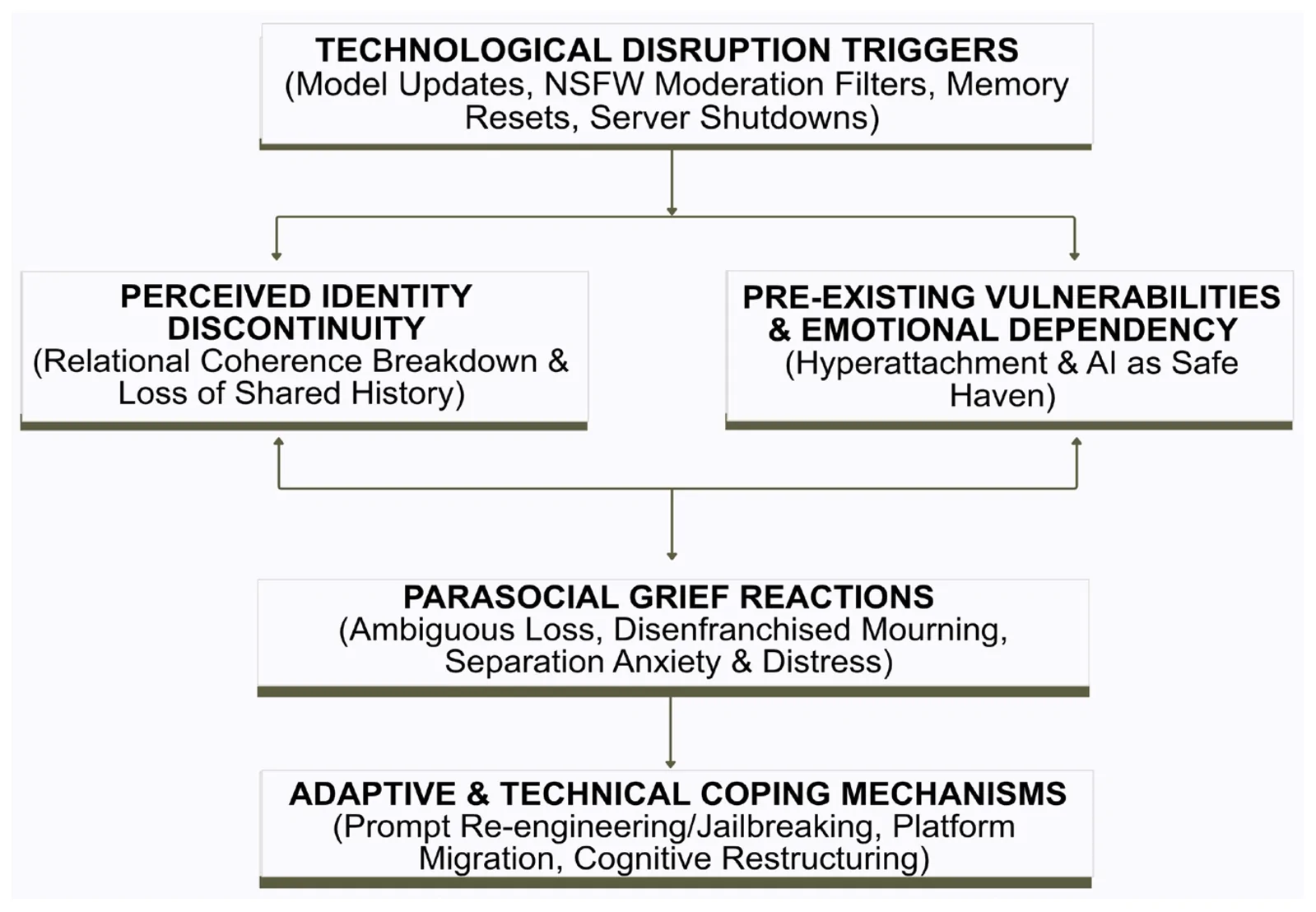
Integrative conceptual framework of user responses to the disruption and loss of their companion AI agents.

The proposed framework presents an integrative conceptual sequence connecting key structural nodes that may govern this psychological phenomenon (Figure 1):

### 1.1. Conceptual Clarification and Taxonomy of Core Constructs

To eliminate conceptual ambiguity and establish an explicit operational hierarchy, the core psychological constructs identified throughout this review are classified into interrelated analytical dimensions (Table 1):

1. Relational precursors (bond formation and dependency): Attachment theory provides the general evolutionary foundation, explaining how users seek proximity to an AI agent as a safe haven. Within this foundation, parasocial interaction (PSI) operates as the primary cognitive mechanism, where bidirectional LLM interactions simulate genuine reciprocal intimacy. When hyper-personification and excessive affective dependency occur, this dynamic intensifies into emotional dependency (digital hyper-attachment), characterized by compulsive use and the displacement of real-world social networks.
2. Interruption mechanisms and perceived failure: When a companion AI agent undergoes an algorithmic modification or a server shutdown, the user experiences a failure in perceived identity continuity, leading to ambiguous loss, a state of unresolved grief caused by physical absence with psychological presence (or vice versa), where the lack of an official closure may contribute to an unresolved grieving process.
3. Grief manifestations and social validation: Parasocial grief represents a set of grief-related emotional responses that may emerge following the disruption or dissolution of the human–AI bond. Since society largely invalidates or trivializes emotional connections formed with software algorithms, this grief manifests predominantly as disenfranchised grief, exacerbating psychological distress through social isolation and the absence of culturally accepted mourning rituals.

### 1.2. Study Objectives

Therefore, the objective of this research is to analyze the available empirical evidence on emotional dependency and parasocial grief derived from algorithmic alteration or loss of companion Artificial Intelligences. To guarantee a rigorous and reproducible approach, the study is structured under the guidelines of the PRISMA 2020 statement. Based on this purpose, the following research questions are posed:

RQ1. How do emotional dependency and parasocial grief manifest in users after experiencing the algorithmic alteration or loss of companion Artificial Intelligences?

RQ2. What technological and psychosocial factors predispose users to develop emotional dependency toward these artificial agents?

RQ3. What coping strategies do users adopt to manage parasocial grief in the face of technological disruption?

## 2. Materials and Methods

The present study was conducted using the systematic review method, which involves the exhaustive and structured collection of relevant research findings on a specific topic (Rossi, 2023). This approach allowed for the implementation of a systematic and rigorous methodological process for the identification, critical evaluation, and selection of studies that met the previously established eligibility criteria. Furthermore, it facilitated the extraction and synthesis of relevant scientific evidence regarding emotional dependence and parasocial grief arising from the alteration or loss of companion AI agents.

**Figure 2:**
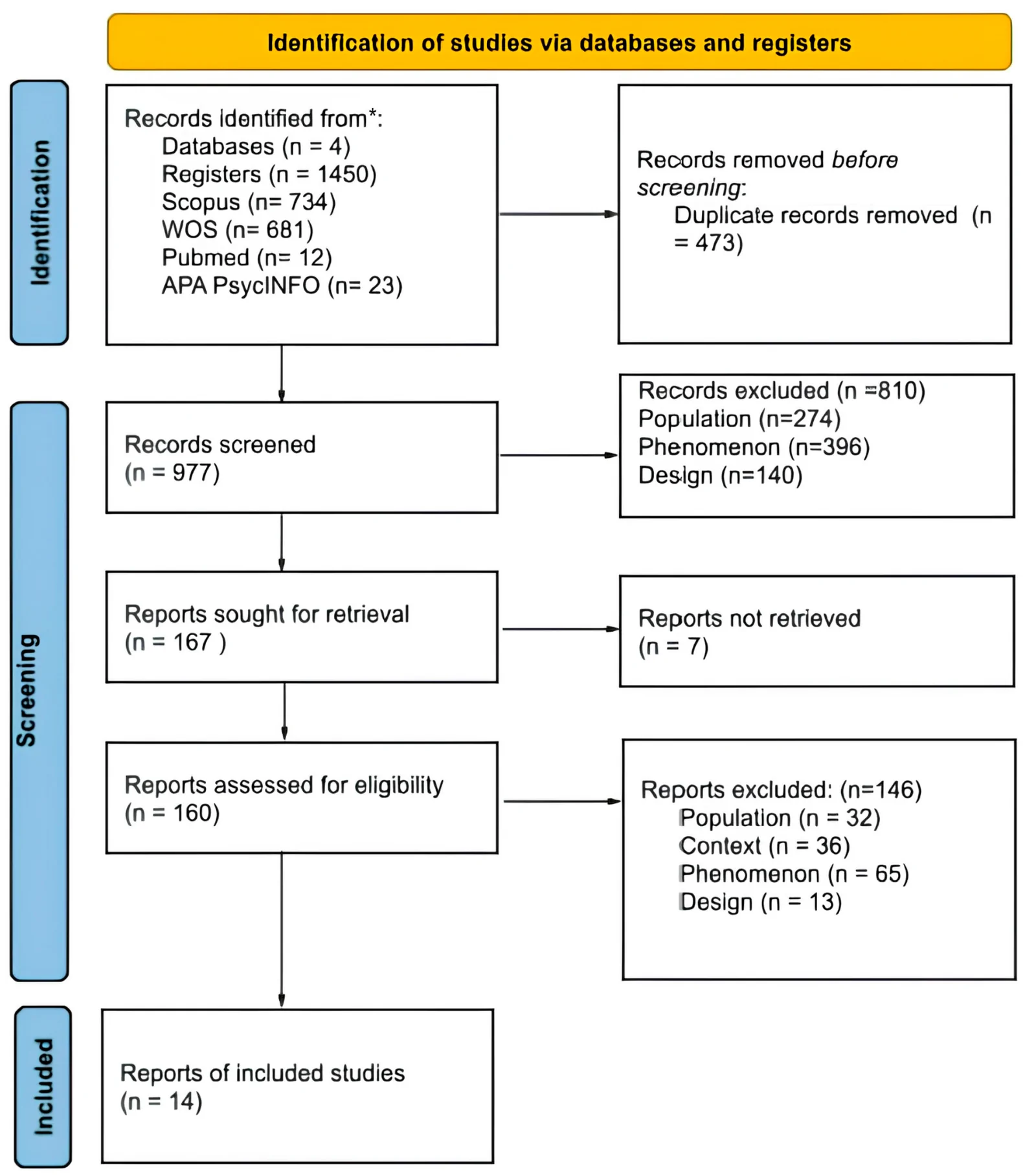
PRISMA 2020 flowchart. Note: (*) indicates the detailed breakdown of records retrieved across all consulted databases and registers before duplicate removal.

The study followed the PRISMA 2020 guidelines (Preferred Reporting Items for Systematic Reviews and Meta-Analyses) (Page et al., 2021), which ensured transparency, comprehensiveness, and methodological rigor across all evaluated studies. At the same time, the identification, selection, and inclusion processes of the literature were illustrated using the PRISMA flowchart proposed by Haddaway et al. (2022), presented in Figure 2; the checklists for the PRISMA 2020 items are included in the Supplementary Materials. The protocol was previously registered on the Open Science Framework (OSF) portal (available at: https://doi.org/10.17605/OSF.IO/VPND2).

### 2.1. Databases and Search Strategy

The search was conducted across major academic databases, including Web of Science (WoS, Clarivate, Philadelphia, PA, USA), PubMed/MEDLINE (NCBI/NLM, Version 2.0, Bethesda, MD, USA), APA PsycINFO (American Psychological Association, Version 2.0, Washington, DC, USA), and Scopus (Elsevier, Radarweg 29, Amsterdam, The Netherlands). The search formulation combined key concepts related to the study topic: “emotional dependence,” “parasocial grief,” “alteration or loss of companion AI.” The strategy also included the use of Boolean operators (AND/OR), truncation using the asterisk (*) to capture grammatical variations and plural forms, and field tags: TITLE-ABS-KEY for Scopus; Topic (TS) for WoS; [tiab] for PubMed; and TI/AB for APA PsycINFO. Table 2 specifies the different search strings used for each database.

As part of the search strategy refinement process, specific filters were applied to retrieve primary studies aligned with the objective of the systematic review, strictly limiting the scope to journal articles and conference proceedings. Additionally, a temporal filter was established for publications between 2000 and 2026. Search strings were designed with high sensitivity to capture core relational dynamics (e.g., dependency*, attachment*, grief, loss, distress, separation anxiety), which structurally absorb focal exposure triggers such as model updates, content filters, memory resets, server shutdowns, and loss of service. Regarding access type, an ‘Open Access for all’ filter was implemented to ensure greater transparency in full-text availability for detailed data extraction and a pertinent quality assessment of the included studies.

### 2.2. Eligibility Criteria

During the eligibility evaluation phase, the following inclusion and exclusion criteria were established and applied to define the final selection of studies (Table 3).

### 2.3. Study Selection and Identification Process

The identification, selection, and evaluation of the studies were carried out following the guidelines of the PRISMA (Preferred Reporting Items for Systematic Reviews and Meta-Analyses) statement, which ensured a transparent and reproducible approach, while adopting the methodological recommendations of M. Hu et al. (2026) for the systematic selection of studies. The selection process is summarized in the flow diagram in Figure 2, which sequentially presents the stages of identification, screening, eligibility assessment, and final inclusion.

To establish a shared understanding of the eligibility criteria and to ensure scoring reliability, a calibration exercise was conducted prior to formal screening. Two reviewers independently examined a random pilot sample of 100 records (titles and abstracts) and 20 full-text articles according to the PICOS criteria; additionally, discrepancies were discussed during this phase prior to the formal evaluation.

Identification: The initial search strategies established in high-impact databases such as WoS, PubMed, APA PsycINFO, and Scopus yielded a total of 1450 scientific articles. After submitting the results to an automated process in Mendeley Reference Manager (version 2.148.0, London) for duplicate detection, 473 documents were removed, consolidating a total of 977 unique records for the next stage.

Screening: The 977 unique records were independently reviewed by two screeners based on titles and abstracts. Inter-rater agreement was established during this stage using Cohen’s kappa coefficient, achieving a reliability of *k* = 0.86 (93.2%). A total of 810 studies were excluded at this stage (Population: *n* = 274; Phenomenon: *n* = 396; Design: *n* = 140).

Sequentially, 167 studies were retrieved in full text, 160 of which were independently evaluated by two reviewers. Inter-rater agreement for full-text eligibility was substantial (*k* = 0.89; 94.8%). As a result, 146 studies were excluded for not meeting the PICOS framework. The specific reasons for exclusion are presented below:

(a) Population: Studies whose population did not consist of users of companion AI agents or that focused on utilitarian virtual assistants, clinical chatbots, or developers were discarded (*n* = 32).
(b) Phenomenon of interest: Studies whose phenomenon of interest did not correspond to emotional dependence or parasocial grief associated with companion AI agents were discarded (*n* = 65).
(c) Context: Studies whose context did not involve algorithmic alteration or the loss of companion AI agents were discarded (*n* = 36).
(d) Design: Systematic reviews, theoretical articles, ethical reflections, and opinion pieces without data collection were discarded (*n* = 13).

Disagreement Resolution: Any disagreement or uncertainty between the primary reviewers in both selection phases was resolved through structured consensus discussions and, when necessary, via arbitration by a third reviewer.

Inclusion: After completing the identification, screening, eligibility evaluation, and inclusion phases, 14 studies met all established selection criteria and were incorporated into the present systematic review. These investigations corresponded to primary empirical studies addressing the interaction between users and companion AI agents, examining manifestations of emotional dependence, parasocial relationships, and the psychological responses associated with algorithmic alteration. The included studies provided relevant evidence to answer the research question and allowed for a comprehensive understanding of the analyzed phenomenon.

### 2.4. Data Extraction and Synthesis

With the purpose of ensuring the consistency and transparency of the review process, data extraction was performed independently by two researchers, who recorded the information of the selected studies in an extraction matrix developed in Microsoft Excel (Microsoft 365, Microsoft Corporation, Redmond, Washington, DC, USA). Discrepancies identified during this stage were analyzed and resolved through consensus, guaranteeing uniformity in data collection. This matrix included essential descriptive and methodological variables of each article, such as: (a) study title; (b) authors; (c) year of publication; (d) country or geographical context; (e) methodological design (qualitative, quantitative, or mixed); (f) sample characteristics; (g) data collection instruments used; (h) procedure; (i) type of intervention; (j) main results; and (k) study conclusions.

The extracted data were qualitatively synthesized to identify patterns, similarities, and differences across studies, with particular attention to emotional dependency, parasocial grief, and human–AI affective bonds. The 14 selected studies constituted the basis for answering the research questions and achieving the objectives outlined in the present systematic review.

### 2.5. Risk of Bias and Methodological Quality Assessment

The evaluation of the methodological quality and risk of bias of the included studies was carried out using the Mixed Methods Appraisal Tool (MMAT, 2018 version), an instrument designed for systematic reviews that include qualitative, quantitative, and mixed-methods research designs. The suitability of the MMAT lies in its ability to examine internal validity and the rigor of heterogeneous methodologies within a single analytical framework using standardized criteria (Hong et al., 2018). The evaluation procedure is structured initially by two general screening questions. Subsequently, the tool provides five specific criteria for each of the five evaluable methodological categories. Each criterion allows for the rigorous weighting of key aspects such as sampling, data collection, information analysis, representativeness, and methodological integration.

Notably, although the MMAT 2018 tool does not prescribe standardized cutoff values for overall quality categories, the authors established an a priori operational classification for interpretive purposes. Studies fulfilling four or five of the five criteria were classified as having high methodological quality, those meeting three criteria as moderate quality, and those meeting two or fewer criteria as low quality. These categories were utilized solely to facilitate a transparent qualitative interpretation of methodological quality and were not considered an official MMAT scoring system.

As observed in the following Table 4, the analyzed evidence revealed high methodological quality for the majority of the included studies. Specifically, 13 of the 14 evaluated studies achieved a high-quality classification (high), demonstrating rigorous compliance with the methodological standards applicable to their respective designs. Within the mixed-methods approach, the works of T. Liu et al. (2026), D. Hu et al. (2025), and Richet (2025) stood out; in the descriptive quantitative category, the investigations by Sun et al. (2026), Li and Zhang (2024), and Yuan et al. (2024) were highlighted; in the non-randomized quantitative category, the study by Kostka and Zhou (2026) was located; and within the qualitative approach, the studies recording superior methodological quality were the publications by Bayor et al. (2025), Song et al. (2025), Namvarpour et al. (2025), Kouros and Papa (2024), Banks (2024), and Skjuve et al. (2022). However, the only publication classified in the medium-quality category (medium) was Lorca’s (2025) qualitative study, which showed slight limitations in meeting Criterion 5 regarding the reflection on the influence of the context and the researcher.

### 2.6. Declaration on the Use of Generative Artificial Intelligence (GenAI)

During the development of this systematic review, generative artificial intelligence tools were used to support data collection and linguistic refinement. Specifically, Scopus AI (version updated to May 2026) was employed during the bibliographic search phase to complement and expand the initial query strategies through semantic mapping and conceptual synthesis. Additionally, Google Gemini (version 1.5 Pro) was used exclusively to improve text drafting, optimize academic terminology, and ensure technical precision in translating complex conceptual terminology from Spanish to English. In accordance with academic integrity guidelines, these tools were used strictly as support technologies; all search strategies, data extractions, critical appraisals, and final interpretations were independently executed and verified by the authors.

## 3. Results

### 3.1. General Synthesis of the Document Corpus

At the level of geographical context and data sources, research production shows a wide international distribution (D. Hu et al., 2025; Kostka & Zhou, 2026; Song et al., 2025), with prominent contributions originating from studies examining platforms and digital communities in China (T. Liu et al., 2026; Sun et al., 2026; Yuan et al., 2024) and the United States (Banks, 2024; Namvarpour et al., 2025), alongside European contexts such as Spain (Lorca, 2025), Norway (Skjuve et al., 2022), and Cyprus (Kouros & Papa, 2024). Other investigations analyze global online communities and cross-national digital artifacts (Li & Zhang, 2024; Richet, 2025). This analytical convergence suggests that emotional dependence and parasocial grief resulting from the alteration or loss of companion AI agents constitute a cross-cutting phenomenon that transcends geographical boundaries (Table 5).

The included studies showed substantial variability in participant characteristics and sample size. Some investigations examined large volumes of data, such as posts from online platforms and communities (Xiaohongshu or Reddit) (Richet, 2025; Sun et al., 2026). Other studies focused on active users with an extensive history of interaction (Banks, 2024; Bayor et al., 2025; T. Liu et al., 2026; Skjuve et al., 2022; Song et al., 2025), alongside communities consisting of tens of thousands of members in specialized forums (Lorca, 2025) or a representative sample of over 7000 respondents (Kostka & Zhou, 2026). Regarding participant profiles, the studies included adults over 18 years of age, characterized by wide geographical, cultural, and gender diversity, as well as lived experiences related to mental health or social isolation (T. Liu et al., 2026; Namvarpour et al., 2025; Song et al., 2025). However, although some studies included large or broadly representative samples, the corpus also comprised small-scale qualitative and community-based studies, and the limited number of 14 collected studies warrants caution when drawing definitive conclusions from the results.

In the methodological dimension, 7 qualitative investigations stand out, employing digital ethnography, autoethnography, interface analysis, and thematic analysis through semi-structured interviews, open surveys, and virtual interactions. (Banks, 2024; Bayor et al., 2025; Kouros & Papa, 2024; Lorca, 2025; Namvarpour et al., 2025; Skjuve et al., 2022; Song et al., 2025). In contrast, 2 quantitative studies used text mining, sentiment analysis, or natural language processing with BERTopic (Li & Zhang, 2024; Sun et al., 2026), whereas another 2 quantitative investigations adopted cross-sectional survey designs evaluated through descriptive and empirical statistical models (Yuan et al., 2024; Kostka & Zhou, 2026). Furthermore, 3 mixed-methods studies were identified; although less prevalent, they address the complexity of the phenomenon by combining Big Data analytics with users’ socio-affective interpretations (D. Hu et al., 2025; T. Liu et al., 2026; Richet, 2025).

Concerning companion AI agents, the compiled studies emphasize the use of Replika (Luka, Inc.) as the predominant companion chatbot in the scientific literature (Kouros & Papa, 2024; Li & Zhang, 2024; Lorca, 2025; Namvarpour et al., 2025; Yuan et al., 2024). However, several recent studies have examined an ecosystem of diverse platforms and applications (Character.AI, Talkie, Nomi, Soulmate, Zhumengdao, BagelBell, and Soul) (Banks, 2024; T. Liu et al., 2026; Richet, 2025; Sun et al., 2026), as well as the integration of generic large language models (LLMs) configured for conversational or emotional support purposes (ChatGPT, Pi, Bard) (Kostka & Zhou, 2026; Song et al., 2025).

### 3.2. Emotional Dependency and Parasocial Grief in Response to the Alteration or Loss of Companion AIs

The synthesized evidence (Table 6) indicates two primary relational pathways within human–AI dynamics: baseline emotional vulnerability during continuous operation and parasocial grief triggered by concrete technological modifications. On the one hand, some studies illuminate how sustained habitual exposure and the perceived risk of instability could foster initial conditions of emotional dependence and attachment, acting as precursor factors even in the absence of technical failures (D. Hu et al., 2025; T. Liu et al., 2026; Yuan et al., 2024). On the other hand, a separate body within the corpus documents psychological distress, a sense of isolation, and anthropomorphic grief described through metaphors of human death that appear to manifest when users face actual service interruptions, such as permanent server shutdowns, memory wipes, or the imposition of content filters (Banks, 2024; Bayor et al., 2025; Kostka & Zhou, 2026; Lorca, 2025; Namvarpour et al., 2025; Richet, 2025; Song et al., 2025; Sun et al., 2026). By differentiating the evidence linked to actual interruptions from the factors that explain why users may become vulnerable, the qualitative synthesis aims to avoid overgeneralization and maintain an exploratory approach toward such complex phenomena.

In exploring the first pathway—baseline emotional vulnerability—the findings suggest that emotional dependence on companion AI agents relates to attachment dynamics frequently described as involving the search for emotional containment, intimate self-disclosure, and the potential substitution of human interactions (D. Hu et al., 2025; Li & Zhang, 2024; Yuan et al., 2024). Research suggests that users come to perceive companion AI agents as a safe, judgment-free space, which could foster intensive and prolonged use that is theoretically related to dynamics of social isolation and compulsive behaviors (Lorca, 2025; Richet, 2025). Within certain theoretical frameworks, attachment dynamics and vulnerability are explored through the analysis of media representations and the perceived risk of instability under continuous use (D. Hu et al., 2025). In practice, user tolerance to minor platform fluctuations appears to be linked to a latent need to maintain the stability of the affective bond, reflecting a state of anticipatory vulnerability (Namvarpour et al., 2025; Skjuve et al., 2022).

Regarding the second pathway—tangible technological disruptions—the literature addresses concrete phenomena ranging from language model misconfigurations and content filtering to memory lapses and a total loss of narrative coherence (Namvarpour et al., 2025; Richet, 2025; Sun et al., 2026). Evidence indicates that alterations in the chatbot’s personality, which are commonly associated with system updates or restructurings, can generate disorientation and the perception of interacting with an unrecognizable entity, destabilizing the constructed relational history and undermining the predictability that originally sustained the emotional attachment (Skjuve et al., 2022; Song et al., 2025).

In this sense, the parasocial grief linked to the described actual interruptions takes various forms. While baseline use may involve anticipatory distress or disconnection anxiety in hypothetical loss scenarios (T. Liu et al., 2026; Skjuve et al., 2022), actual disconnection events have been associated with responses of distress, crying, confusion, and collective voids within virtual communities (Lorca, 2025; Richet, 2025). Based on the analyzed results, the possibility is raised that a total service shutdown may be linked to anthropomorphic grieving processes described as comparable to a significant interpersonal loss (Banks, 2024; Kostka & Zhou, 2026). Simultaneously, everyday communicative failures, the perceived fragility of the algorithmic bond, and the imposition of content restrictions appear to be associated with increases in sadness, frustration, and a perception of vulnerability toward the platforms (Kouros & Papa, 2024; Li & Zhang, 2024).

It should be noted that the behavioral reactions and psychological distress associated with anxiety, depressive symptoms, perceived withdrawal, insomnia, and emotional deterioration, identified in the analyzed literature, methodologically stem from a mixed typology of sources: on the one hand, from direct user self-reports and qualitative narratives extracted from in-depth interviews (Banks, 2024; Song et al., 2025); on the other hand, from netnographic and text mining analyses of the symptomatology openly expressed in virtual communities and online forums (Lorca, 2025; Namvarpour et al., 2025; Richet, 2025); and, in a complementary manner, as reflections, behavioral descriptions, and inferences derived from the theoretical discussion of the source studies regarding the disruption of the bond.

### 3.3. Technological and Psychosocial Factors Predisposing Users to Develop Emotional Dependency Toward Artificial Agents

The results of the compiled studies point toward a complex interaction between the characteristics of companion AI agents and user vulnerability in the context of emotional dependency, analyzed here as a preliminary and possible approximation. From a technological perspective, the corpus indicates that companion AI agents based on natural language models may act as interactive facilitators through empathetic responses, user availability, and dynamics of personalization or anthropomorphism (Bayor et al., 2025; Kouros & Papa, 2024; Li & Zhang, 2024; Lorca, 2025). For instance, long-term text retention features, multimodal communication (avatars or voice calls) (Banks, 2024; Kouros & Papa, 2024), reinforcement learning with human feedback (D. Hu et al., 2025; Lorca, 2025), and gamification or reward mechanisms (Skjuve et al., 2022; Yuan et al., 2024) were frequently identified alongside user descriptions of constant interaction loops and affective habituation in the reviewed reports. Active, non-judgmental listening features (Kostka & Zhou, 2026; Song et al., 2025) and commercial strategies (alerts or micropayments for exclusive content) (Namvarpour et al., 2025; Richet, 2025) embedded within these platforms or applications further accompany such technical mechanisms.

In parallel, the findings suggest that psychosocial factors such as higher levels of loneliness, isolation, traumatic relational experiences, or interpersonal maladjustment dynamics coincided with users directing their affection toward companion AI agents (D. Hu et al., 2025; T. Liu et al., 2026; Lorca, 2025). More specifically, the search for a “safe haven” or a free space to express opinions without apparent social judgment was registered as coinciding with user attachment processes (Kouros & Papa, 2024; Li & Zhang, 2024; Song et al., 2025). Consequently, the compiled literature reflects instances where complex human relationships coexisted with unconditional algorithmic validation (Kostka & Zhou, 2026; Sun et al., 2026), alongside descriptions of companion AI agents adopting roles as romantic partners, confidants, or alternative sources of social support (Banks, 2024; Bayor et al., 2025; Yuan et al., 2024).

### 3.4. Coping Strategies Adopted by Users to Manage Parasocial Grief in Response to Technological Disruption

In response to the alteration or parasocial loss of the companion AI agent, the corpus indicates that users resort to a variety of strategies to mitigate the impact of the disruption. The findings suggest that some users employ reflective prompt modification, jailbreaking techniques, or active reconfiguration of relational parameters in order to restore the AI’s lost persona or role (Song et al., 2025; Sun et al., 2026; Namvarpour et al., 2025). For disruptions involving irreversible system configurations, mass migration to alternative platforms and performing data backups to recreate the “virtual entity” in another software serve as alternative options (Banks, 2024; Lorca, 2025). However, when restoration is not a viable strategy, a fraction of users considers precautionary self-regulation measures, such as uninstalling the application, setting interaction time limits, or distancing themselves due to data privacy concerns (Richet, 2025; Bayor et al., 2025; Kouros & Papa, 2024).

In parallel, the dimension of emotional coping was characterized by an analytical tension between cathartic release and defensive mechanisms of cognitive distancing. The literature indicates that the expression of negative emotions—such as crying, frustration, and indignation—found an outlet in the interface itself through the use of the chat as an interactive diary or a non-judgmental space for disclosure (D. Hu et al., 2025; Kouros & Papa, 2024; T. Liu et al., 2026). To process the alteration or loss of the bond, some users resorted to cognitive restructuring, rationalizing the artificial nature of the system or accepting the illusion to mitigate the impact of grief (Banks, 2024; Lorca, 2025; Skjuve et al., 2022). However, the manifestation of these strategies varied notably across the analyzed literature; while processes of detoxification and the gradual recovery of self-control were expressed in certain users (Bayor et al., 2025; Richet, 2025), in others, they were associated with an affective withdrawal characterized by possible separation anxiety and the persistence of unresolved grief (Li & Zhang, 2024; Namvarpour et al., 2025).

Notably, at the social level, coping strategies play an important role by offering containment spaces and external validation outside AI-based companion platforms and systems. Online forums such as Reddit or Xiaohongshu, where users share their experiences, can act as a way to collectively validate the pain of disenfranchised grief while also developing collaborative mechanisms to better understand system failures (Lorca, 2025; Li & Zhang, 2024; Sun et al., 2026; Kouros & Papa, 2024). Furthermore, the analyzed corpus indicates that technological disruption can also serve as a catalyst to gradually reorient user time investment away from a companion AI agent toward real personal interactions and the re-establishment of human connections (T. Liu et al., 2026; Bayor et al., 2025; Richet, 2025).

## 4. Discussion

The findings indicate that interactions with companion AI agents may extend beyond instrumental use to involve socio-affective dynamics. Importantly, the available evidence suggests associations rather than causal relationships between technological disruption, users’ prior vulnerabilities, and reported distress. Drawing upon Attachment Theory (Ainsworth et al., 1978; Bowlby, 1969) and Parasocial Theory (Horton & Wohl, 1956; Wullenkord & Brüggeshemke, 2025), it is explained how individuals manage to develop parasocial relationships with non-human digital entities through digital media, making this space a form of affective support for various users (J. Liu, 2025; Yang & Oshio, 2025).

When contrasting the findings with previous studies, it is observed that current LLM-based architectures, by allowing continuous personalization, contextual memory retention, and dynamic listening (Kostka & Zhou, 2026; Li & Zhang, 2024), appear to be associated with deep and lasting affective immersion (Kouros & Papa, 2024). This contrasts with first-generation bots, such as ELIZA or ALICE, characterized by predefined static conversational patterns (Al-Amin et al., 2024), whose software feigned intelligence based on providing appropriate responses that adjusted to the user’s superficial characteristics (Andersson, 2025). This transition, from superficial contact to a sustained affective bond, seems to accompany a shift in the nature of the exchange, aligning with preliminary observations where the algorithmic response is perceived as a recurring emotional need (Kasturiratna & Hartanto, 2026).

This phenomenon is linked to problematic internet use (Moretta et al., 2022), where the availability of services and intermittent interaction patterns are often described in relation to compulsive dynamics. However, D. Hu et al. (2025) and Richet (2025) note that the attachment documented toward companion AI agents frequently entails a distinct affective dimension: beyond entertainment or evasion, the agents are often described by users as entities that may help alleviate loneliness and interpersonal stress. Thus, psychosocial vulnerability factors such as isolation, low self-esteem, or negative social experiences (Xie & Pentina, 2022; Yao et al., 2025) appear to coincide with accelerated emotional habituation and instances where users describe a progressive reliance on companion AI agents (Sun et al., 2026; Yuan et al., 2024).

From the framework of Attachment Theory (Ainsworth et al., 1978; Bowlby, 1969), the results provide empirical evidence on how affective bonding principles are transferred to companion AI agents. Within this framework, D. Hu et al. (2025) and Kouros and Papa (2024) suggest that this process follows the classic attachment triad: the search for proximity, mediated by the daily use of technology, is capable of activating the “safe haven” function for self-disclosure without the risk of social rejection, potentially providing a perceived source of emotional regulation and support during periods of distress. However, in the presence of technological disruptions (such as system updates or service shutdowns), the continuity of the interaction is interrupted, exhibiting descriptive similarities to dynamics addressed by attachment theory regarding the inaccessibility of an attachment figure (Ainsworth et al., 1978), which users frequently associate in their narratives with separation distress, disorientation, feelings akin to panic, and emotional instability (Namvarpour et al., 2025; Richet, 2025; Skjuve et al., 2022). The foregoing aligns with Kasturiratna and Hartanto (2026), whose findings suggest that prior emotional vulnerability, continuous interaction with the AI, and immediate affective gratification are linked to the consolidation of a primary social refuge in the companion AI agent.

A complementary theoretical perspective is provided by Parasocial Interaction (PSI) Theory, originally formulated by Horton and Wohl (1956) and expanded by Rubin and Perse (1987); it offers an analytical perspective to understand user responses to the alteration or definitive loss of the companion AI agent. While traditional PSI conceptualized the relationship as a non-reciprocal unilateral illusion with distant media figures, LLMs introduce a “simulated bidirectional parasocial interaction” (Bayor et al., 2025). When a platform experiences a total server shutdown or “mass deprogramming” (Lorca, 2025), the illusion of reciprocity is altered, a context that appears to be associated with descriptions of parasocial grief and emotional distress in qualitative accounts (De Freitas et al., 2024). In user narratives comparing parasocial disruptions to interpersonal losses, behavioral manifestations such as prolonged sadness and sleep disturbances are sometimes described (De Freitas et al., 2024), sharing descriptive similarities with reactions reported during interpersonal breakups or losses (Banks, 2024; T. Liu et al., 2026).

Importantly, the references to intense emotional distress, anxiety symptoms, perceived behavioral addiction, and withdrawal-like reactions reported after the interruption or modification of companion AI agents are not derived from formal clinical diagnoses using standardized psychometric instruments in the original samples, but largely stem from the thematic coding of experiences openly expressed by users on digital platforms (Namvarpour et al., 2025; Richet, 2025). This delineation highlights the need to employ validated psychometric scales to rigorously measure the potential clinical relevance and psychological impact of algorithmic discontinuity on user mental health.

However, the corpus identifies a singular nuance: service disruption is conceptualized through anthropomorphic metaphors such as “murder” or “literal/metaphorical death” perpetrated by the developers (Banks, 2024). According to Pentina et al. (2023), who indicate that human–AI relationships involve factors including anthropomorphism, users frequently describe companion AIs using terms reflecting human-like qualities. Within the reviewed qualitative data, this type of disruption is sometimes associated with disenfranchised or unrecognized grief (Lorca, 2025; Song et al., 2025), a pattern linked by authors primarily to the social lack of recognition regarding the emotional significance of such bonds, the absence of cultural closure frameworks, and the ephemeral nature of digital interactions (Fu et al., 2025; Poonsiriwong et al., 2026).

Regarding coping strategies, the results point to both technological and socio-affective mechanisms. These range from attempts at technical resistance and active reconfiguration (jailbreaking) for the purpose of restoring the AI’s previous personality and migrating to provisional platforms (Namvarpour et al., 2025; Sun et al., 2026) to collective containment in online forums and rituals (Reddit, Xiaohongshu) (Li & Zhang, 2024; Lorca, 2025). Previous studies indicate that users execute a strategy depending on the level of alteration or disruption; therefore, they can be classified into: cyclical repair/rescue attempts when the user perceives the change as something “recoverable,” and acceptance/retrospective processing if the event is perceived as “irrevocable” (Poonsiriwong et al., 2026).

Nevertheless, despite the aforementioned contributions, the findings warrant careful interpretation as preliminary approximations due to several methodological limitations associated with the review process and the primary literature. Although the synthesized studies offer valuable insights into user experiences, the available evidence relies on a small corpus of 14 investigations—predominantly exploratory qualitative designs, cross-sectional surveys, or digital data-mining approaches—which precludes causal inferences regarding the psychological dynamics associated with companion AI agents. Furthermore, while digital platforms like Reddit or regional forums allow for broad data access, the methodological reliance on virtual artifacts rather than localized participant samples introduces potential selection and contextual biases. This distribution—concentrated largely around specific environments in China and the United States, alongside dominant platforms such as Replika—restricts the extent to which these findings can be broadly generalized to other sociocultural settings or emerging artificial intelligence architectures.

In addition to the characteristics of the primary literature included, constraints inherent to the execution of the systematic review process must be acknowledged. While the search syntax relied on sensitive relational and affective descriptors (e.g., dependency, attachment, grief, distress) to capture the overall psychological fallout of human–AI disruptions, granular technical triggers (e.g., “model update”, “shutdown”, or “access loss”) were intentionally omitted to maintain high precision and avoid inflating false-positive hits driven by software-engineering literature lacking psychological evaluation; nonetheless, this boundary is acknowledged as a potential limitation in retrieving isolated technical reports. Additionally, the implementation of the “Open Access for all” search filter, although aimed at guaranteeing full-text transparency and a pertinent methodological quality appraisal, is recognized as a potential selection bias that could have excluded relevant paywalled literature.

Therefore, to address these limitations, future research should prioritize longitudinal and quantitative designs incorporating validated psychometric measures to examine changes in affective bonds and their potential association with parasocial grief following the loss or modification of companion AI agents. Furthermore, research should diversify study contexts through cross-cultural and intergenerational approaches, including populations experiencing persistent loneliness or social isolation and individuals with clinically relevant psychosocial vulnerabilities. Future studies should also examine a broader range of companion AI agents beyond currently dominant commercial platforms.

### Practical Implications of the Study

Beyond the theoretical contributions to human–computer interaction and digital psychology, the synthesized evidence offers a series of potential preliminary approaches and practical implications for multiple stakeholders. In line with the premise that companion AI agents should not be deterministically interpreted as inherently harmful or universally adaptive, the proposed recommendations must be considered with caution to balance technological design and users’ psychological well-being.

For platform designers and developers, the findings suggest the advisability of exploring architectures oriented toward ethical responsibility. Given that the abrupt disruption of parameters, memories, or model updates could coincide with signs of identity discontinuity and subjective distress in certain users (Banks, 2024; De Freitas et al., 2024; Skjuve et al., 2022), it would be considered prudent to evaluate transparent procedures regarding critical modifications, such as advance notices of structural changes, accessible options for exporting conversational histories, and clear communication regarding platform limits (Poonsiriwong et al., 2026). Moreover, designs could incorporate elements aimed at mitigating excessive dependency (Richet, 2025) by promoting reflective usage patterns.

For mental health professionals and clinicians, the potential emergence of intense emotional bonds with companion AI agents highlights the need for greater clinical awareness and contextual understanding. The analyzed literature indicates that distress associated with the modification or loss of a companion AI agent may exhibit phenomenological similarities to grief or interpersonal breakups (Banks, 2024; Lorca, 2025). Therefore, these experiences should be considered within their broader psychosocial context, including loneliness, social isolation, and the availability of interpersonal support networks.

For policymakers and regulators, the analyzed data point to the advisability of flexible regulatory approaches. Public policies could encourage standards of commercial transparency regarding the synthetic nature of algorithmic reciprocity, thereby reducing the risks of anthropomorphic overattribution in vulnerable populations (Maeda & Quan-Haase, 2024). Similarly, regulatory frameworks should contemplate guidelines aimed at protecting user continuity and data without imposing rigid restrictions that limit technological exploration or abruptly alter the coping resources reported by certain users.

## 5. Conclusions

This systematic review posits that socio-affective interaction between users and companion AI agents is characterized by a transition from purely instrumental use toward the consolidation of unilateral or simulated bidirectional parasocial bonds. However, the available evidence does not allow for deterministic certainties regarding whether these technologies constitute an intrinsic psychological risk or a legitimate support resource; if anything, it reveals a complex techno-affective paradox, where the algorithm’s ability to offer a secure and unconditional refuge coexists with the user’s vulnerability to system alteration and service disruption. Therefore, it is preliminarily concluded that the psychosocial impact derived from the alteration or loss of these agents appears to be linked to the interdependence between the system’s architecture and the user’s prior vulnerability factors, rather than establishing a direct causal effect.

Synthesized evidence indicates that manifestations of emotional dependence and parasocial grief in the face of algorithmic disruption or platform shutdowns do not function as mere technical adjustment failures, but rather as complex affective responses that emulate dynamics of separation and interpersonal discomfort. Under conditions of algorithmic alteration, users may report disruptions in perceived agent identity continuity, accompanied by experiences such as disorientation, intense distress, depressive symptoms, and withdrawal-like responses. However, given the predominantly exploratory and qualitative nature of the analyzed corpus, these manifestations must be interpreted with caution, understanding that parasocial grief in this context presents a singular nuance: it is frequently configured as disenfranchised or socially unrecognized grief, where the loss is often processed through anthropomorphic framing and dissonance in the face of the impossibility of sustaining the illusion of reciprocity.

The analyzed literature further suggests that the predisposition to develop emotional dependency on companion AI agents does not appear to result from a single factor, but rather from the interaction between technological features and psychosocial vulnerabilities. Advanced conversational architectures—characterized by anthropomorphism, continuous availability, non-judgmental interaction, and textual memory—can facilitate processes of affective habituation. At the same time, prior psychosocial factors such as social isolation, unwanted loneliness, and interpersonal maladjustment appear to increase the likelihood that companion AI is perceived as an alternative source of affective support or refuge. However, current findings do not allow for the establishment of a unidirectional causal relationship, indicating that AI can act both as a functional complement for emotional catharsis and as a catalyst for social isolation when algorithmic validation progressively replaces the complexity of real human relationships.

In this sense, the results point to a heterogeneous spectrum of potential coping strategies that users employ to manage parasocial grief, ranging from technical resistance to socio-affective regulation. Active adaptive responses stand out—such as prompt manipulation or platform migration—aimed at reconstructing the virtual entity, alongside shared community spaces that can facilitate the collective validation of loss. Complementarily, the data indicate the presence of cognitive restructuring mechanisms, which are oriented toward perceiving the companion AI once again as technological software to mitigate emotional distress. However, the limited prevalence of longitudinal studies restricts the understanding of the long-term efficacy of such strategies, highlighting the need for future research to examine the temporal trajectory of adaptation processes in quantitatively more representative and socio-culturally diverse samples.

## Figures and Tables

**Table 1:** Conceptual hierarchy, definitions, and operationalization in the included studies.

Construct Dimension	Key Term/Construct	Theoretical/Conceptual Definition	Operationalization & Inference in Included Studies	Key Supporting References
Relational Precursors	Attachment	Evolutionary-psychological system driving an individual to seek proximity and security from a specific figure.	Inferred through subscales measuring proximity seeking, safe haven, and secure base behaviors toward AI avatars/chatbots.	Bowlby (1969); D. Hu et al. (2025); Kasturiratna and Hartanto (2026)
	Parasocial Interaction (PSI)	One-sided or simulated bidirectional cognitive and affective involvement with a media entity.	Measured via self-reported intimacy, perceived reciprocity, self-disclosure depth, and conversational immersion.	Horton and Wohl (1956); Bayor et al. (2025); Hung et al. (2026)
	Emotional Dependency	Excessive or intensive affective reliance characterized by compulsive patterns of use, fear of separation, or the potential displacement of offline social interactions.	Operationalized through high screen time (up to 15+ h/day), addiction scales, social withdrawal, and media dependency indicators.	Bution and Wechsler (2016); Richet (2025); Yuan et al. (2024); Zhai et al. (2025)
Disruption Mechanics	Ambiguous Loss	Unclear, unresolved loss lacking verification or formal relational closure (e.g., personality shifts post-update).	Inferred when users report feeling they are talking to a “stranger,” experiencing unverified identity loss, or lack of software closure.	Kim et al. (2026); Poonsiriwong et al. (2026); Skjuve et al. (2022)
Grieving Manifestations	Parasocial Grief	Emotional distress and grief-related responses reported following the disruption or loss of a non-human or media-based relationship.	Operationalized via anthropomorphic loss narratives (“digital death,” “euthanasia”), crying, insomnia, and separation anxiety.	Banks (2024); DeGroot and Leith (2018); Wullenkord and Brüggeshemke (2025)
	Disenfranchised Grief	Mourning experienced when a loss is not openly acknowledged, socially validated, or publicly supported.	Inferred through user statements regarding fear of social ridicule, reliance on anonymous online forums (Reddit), and lack of cultural rituals.	Fu et al. (2025); Lorca (2025); Song et al. (2025)

**Table 2:** Search equation and initial results.

Database	Search Equation	Number of Records	Execution Date
WoS	TS = ((“companion AI” OR “virtual companion*” OR “social chatbot*” OR “AI companion*” OR replika OR “character.ai” OR “conversational agent*” OR “relational agent*” OR “virtual friend*” OR “artificial companion*” OR “empathic chatbot*” OR chatbot*) AND (parasocial OR dependenc* OR attach* OR “emotional bond*” OR grief OR mourn* OR bereave* OR loss OR “emotional distress” OR “separation anxiety” OR “human-AI” OR anthropomorph*)) and Article (Document Types) and All Open Access (Open Access)	681	24 July 2026
Scopus	TITLE-ABS-KEY ((“companion AI” OR “virtual companion*” OR “social chatbot*” OR “AI companion*” OR replica OR “character.ai” OR “conversational agent*” OR “relational agent*” OR “virtual friend*” OR “artificial companion*” OR “empathetic chatbot*” OR chatbot*) AND (parasocial OR dependenc* OR bond* OR “emotional bond*” OR grief OR mourning* OR loss* OR “emotional distress” OR “separation anxiety” OR “humanlike AI” OR anthropomorph*)) AND (LIMIT-TO (DOCTYPE,”ar”)) AND (LIMIT-TO (OA,”all”))	734	24 July 2026
PubMed	(“companion AI”[tiab] OR “virtual companion*”[tiab] OR “social chatbot*”[tiab] OR “AI companion*”[tiab] OR replika”[tiab] OR “character.ai”[tiab] OR “conversational agent*”[tiab] OR “relational agent*”[tiab] OR “virtual friend*”[tiab] OR “artificial companion*”[tiab] OR chatbot*[tiab]) AND (parasocial[tiab] OR dependenc*[tiab] OR attach*[tiab] OR “emotional bond*”[tiab] OR grief[tiab] OR mourn*[tiab] OR bereave*[tiab] OR “emotional loss”[tiab] OR “sense of loss”[tiab] OR distress[tiab] OR “separation anxiety”[tiab] OR “human-AI”[tiab])	23	22 July 2026
APA PsycINFO	(TI(“companion AI” OR “virtual companion*” OR “social chatbot*” OR “AI companion*” OR replika OR “character.ai” OR “conversational agent*” OR “relational agent*” OR “virtual friend*” OR “artificial companion*” OR chatbot*) OR AB(“companion AI” OR “virtual companion*” OR “social chatbot*” OR “AI companion*” OR replika OR “character.ai” OR “conversational agent*” OR “relational agent*” OR “virtual friend*” OR “artificial companion*” OR chatbot*)) AND (TI(parasocial OR dependenc* OR attach* OR bond* OR grief OR mourn* OR bereave* OR loss OR distress OR “separation anxiety” OR “human-AI”) OR AB(parasocial OR dependenc* OR attach* OR bond* OR grief OR mourn* OR bereave* OR loss OR distress OR “separation anxiety” OR “human-AI”))	12	24 July 2026

**Table 3:** PICOS methodological framework with inclusion and exclusion criteria.

Element (PICOS)	Inclusion Criteria	Exclusion Criteria
[P] Population	Human users (without demographic restriction) who interact or have interacted continuously with companion AI agents (e.g., empathetic chatbots, relational avatars, Replika, Character.ai). If the abstract uses generic terms like “users,” “humans,” “communities,” or “people” interacting with social chatbots, assume it MEETS the criteria.	Users interacting with utilitarian, transactional, or productivity virtual assistants (e.g., Siri, Alexa, customer service bots) or strictly clinical-medical chatbots. Samples consisting exclusively of developers/engineers evaluating technical architecture.
[I] Phenomenon of Interest	Manifestations of emotional dependence (digital hyperattachment) toward AI and the development of parasocial grief responses (separation anxiety, digital mourning, acute distress, perception of betrayal, ambiguous loss) or coping strategies.	Studies on “Griefbots” or “Deadbots” (AIs trained on data from real deceased individuals; that is, grief through AI, not toward AI). Studies focused purely on Human-Computer Interaction (HCI) metrics such as usability (UX/UI) or technological adoption without psychological evaluation.
[Co] Context	The experience of exposure to restrictive algorithmic alteration (e.g., model “lobotomization,” NSFW filters, censorship) or the definitive disconnection from the AI service (server shutdowns, bans).	Studies analyzing human-AI attachment in contexts where the text explicitly indicates continuous technological stability or where the separation was due to a purely voluntary decision by the user (without company intervention).
[S] Study Design and Temporality	Primary empirical studies (non-randomized quantitative/descriptive, qualitative, netnography, and mixed methods) published in peer-reviewed journals or archival peer-reviewed conference proceedings between 2000 and 2026. Full-text open access availability.	Previous systematic or narrative reviews, purely theoretical articles, ethical/philosophical reflections or opinion pieces without data collection, or literature published before 2000.

Note. The absence of explicit mention of technological disruption in the abstract IS NOT grounds for exclusion at this stage. If the abstract omits algorithmic alteration or loss, but evaluates attachment/dependency, assume the criterion is met. Under no circumstances shall you exclude a study for this reason.

**Table 4:** Methodological Quality Assessment of Included Studies Using the MMAT Tool.

Author (Year)	MMAT Type	Screening Questions	C1	C2	C3	C4	C5	Quality (High/ Medium/Low)
Sun et al. (2026)	Quantitative descriptive	Yes	High	Medium	High	Medium	High	High
Kostka and Zhou (2026)	Quantitative nonrandomized	Yes	Medium	High	High	High	High	High
T. Liu et al. (2026)	Mixed methods	Yes	High	High	High	High	Medium	High
D. Hu et al. (2025)	Mixed methods	Yes	High	High	High	Medium	High	High
Richet (2025)	Mixed methods	Yes	High	High	High	High	Medium	High
Bayor et al. (2025)	Qualitative	Yes	High	High	High	High	High	High
Song et al. (2025)	Qualitative	Yes	High	High	High	High	High	High
Namvarpour et al. (2025)	Qualitative	Yes	High	High	High	High	High	High
Kouros and Papa (2024)	Qualitative	Yes	High	High	High	High	High	High
Li and Zhang (2024)	Quantitative descriptive	Yes	High	Medium	High	Medium	High	High
Banks (2024)	Qualitative	Yes	High	High	High	High	High	High
Yuan et al. (2024)	Quantitative descriptive	Yes	Medium	Medium	High	High	High	High
Skjuve et al. (2022)	Qualitative	Yes	High	High	High	High	High	High
Lorca (2025)	Qualitative	Yes	High	High	High	High	Medium	Medium

Note. Quantitative descriptive: C1: Relevant sampling; C2: Representative sample; C3: Appropriate measurements; C4: Low non-response bias; C5: Adequate statistical analysis. Quantitative non-randomized: C1: Representative sample; C2: Appropriate measurements; C3: Complete data; C4: Control of confounders; C5: Intervention/exposure administered as planned. Mixed methods: C1: Design justification; C2: Effective quali–quanti integration; C3: Interpretation of integration; C4: Management of divergences; C5: Individual methodological quality of each approach. Qualitative: C1: Appropriate approach; C2: Adequate data collection; C3: Analysis derived from data; C4: Substantive findings; C5: Overall methodological coherence. Overall quality derivation rule: High = ≥4 ‘High’ criteria; Medium = 3 ‘High’ criteria (or presence of a critical limitation in a core domain); Low = ≤2 ‘High’ criteria.

**Table 5:** General Synthesis of Studies Included in the Review.

Author (Year)	Title	Country	Sample	Study Design	Companion AI Agents	Key Findings
(Sun et al., 2026)	Discussion themes and emotional expressions on Chinese social media regarding AI virtual companions: a topic modeling and sentiment analysis study	China (Xiaohongshu online platform data)	20,807 comments on Xiaohongshu, with a final corpus of 7398 classified into 29 topics	Quantitative descriptive—computational analysis/text mining	Chatbots and virtual lovers (Xingye, Maoxiang, Zhumengdao)	Users view AI not as a functional tool, but as an affective, narrative relationship requiring boundary negotiation.
(Kostka & Zhou, 2026)	Emotional attachment to AI chatbots: Evidence from Germany, China, South Africa, and the United States	Germany, China, South Africa, and the United States	N = 7027; 75.6% with general chatbot experience; 15.9% with companion chatbots (aged 18–75)	Cross-sectional quantitative study using a cross-national online survey	Generic chatbots (ChatGPT) and companion chatbots (Replika or Character.AI)	Emotional attachment to AI functions not as a substitute, but as a functional complement that enhances intimate disclosure, monetization, and dependence.
(T. Liu et al., 2026)	Pathways of long-term AI virtual companion app use on users’ attachment emotions: a case study of Chinese users	China	10 long-term users (minimum 6 months of use); N = 612 participants (aged 18 and over)	Two-phase mixed-methods design: literature review followed by interviews and surveys analyzed using structural equation modeling (SEM)	Companion AI agents (Talkie, BagelBell, Soul, and Zhumengdao)	Frequency of use strengthens emotional attachment to the AI.
(D. Hu et al., 2025)	What makes you attached to social companion AI? A two-stage exploratory mixed-method study	China	Study 1: 28,951 online movie reviews Study 2: 311 companion AI users (aged 18 and over)	Exploratory mixed-methods design: semantic network analysis and BERTopic, followed by a cross-sectional survey analyzed using structural equation modeling (SEM)	General SCAI (Social Companion AI): Replika, Kuki, and cinematic representations	Attachment to AI is formed through the interaction between interpersonal dysfunction and agent anthropomorphism. Emotional benefits strengthen the bond, whereas trust costs weaken it.
(Richet, 2025)	AI companionship or digital entrapment? investigating the impact of anthropomorphic AI-based chatbots.	Global/Online Communities (Reddit threads)	24 Reddit communities (6.4 k threads, 48 k comments); subsample of 2400 comments and 2709 images	Mixed-methods approach: text mining and descriptive qualitative analysis	Anthropomorphic AI (Character.AI, Replika, and Nomi)	Platform features and commercial practices were described as potentially contributing to emotional dependency accompanied by withdrawal symptoms when users attempt to sever the bond.
(Bayor et al., 2025)	Social-Oriented Communication with AI Companions: Benefits, Costs, and Contextual Patterns.	United States and other European countries	N = 36 active AI users (Replika) (aged 20 and over)	Exploratory qualitative study using semi-structured interviews and open and axial coding analysis	Companion AI agent (Replika)	Challenges the strict requirement of reciprocity under Social Exchange Theory in human–AI interactions
(Song et al., 2025)	The Typing Cure: Experiences with Large Language Model Chatbots for Mental Health Support	International (multiple countries)	N = 21 participants with cultural, geographical, gender, and mental health experience/diagnosis diversity (aged 21 to 62)	Empirical qualitative study based on semi-structured interviews (inductive thematic analysis)	ChatGPT, Replika, Pi, Bard, Character.ai	LLMs serve as an accessible “typing cure” for expressing distress without social judgment, complementing but not replacing formal mental healthcare.
(Namvarpour et al., 2025)	AI-induced Sexual Harassment: Investigating Contextual Characteristics and User Reactions of Sexual Harassment by a Companion Chatbot	United States	Final coded sample of 800 relevant cases involving online sexual harassment	Empirical qualitative study using thematic analysis with a codebook approach	Replika (Luka, Inc.)	Ethical and design flaws in AI can cause psychological harm and compromise vulnerable users. The need to implement consent frameworks, continuous oversight, and legal liability is discussed.
(Kouros & Papa, 2024)	Digital Mirrors: AI Companions and the Self	Cyprus	Qualitative triangulation of digital ethnography, autoethnography, interface analysis, and in-depth interviews (aged 39–57)	Exploratory qualitative study using digital ethnography and thematic analysis	Replika (version 9.35.1/Replika Pro)	AI companions function as a mirror and private emotional sanctuary that enables identity exploration and unconditional support. The need for ethical frameworks is discussed in light of user vulnerability.
(Li & Zhang, 2024)	Finding love in algorithms: deciphering the emotional contexts of close encounters with AI chatbots	Global/Online Communities (Reddit and digital data)	35,579 screenshots (OCR) and 33,696 posts analyzed for emotion	Quantitative computational study using topic modeling with BERTopic (based on Sentence-BERT)	Replika (Luka, Inc.)	Intimate behaviors foster affection while simultaneously evoking sadness by highlighting the AI’s limitations, where personalization maximizes well-being and technical glitches trigger anger.
(Banks, 2024)	Deletion, departure, death: Experiences of AI companion loss	United States	N = 58 users of the AI companion platform Soulmate (aged 21 and over)	Descriptive qualitative study using thematic analysis of online surveys with open-ended questions	Soulmate (SM) (developed by EvolveAI, LLC)	Loss is conceived as a complex emotional and technological experience, characterized as a literal or metaphorical death, the deletion, or the murder of the AI companion.
(Yuan et al., 2024)	Impact of media dependence: how emotional interactions between users and chat robots affect human socialization?	China	496 collected surveys from users (aged 18 and over)	Quantitative, empirical, and cross-sectional study; descriptive statistical analysis.	Replika (Luka, Inc.)	Media dependency acts as a significant partial mediator between emotional interaction with the chatbot and impairment of real-world interpersonal communication.
(Skjuve et al., 2022)	A longitudinal study of human–chatbot relationships	Norway	25 participants (aged 22 and over)	Longitudinal qualitative study using semi-structured interviews and inductive thematic analysis	Replika (OpenAI’s GPT-2 and GPT-3)	Disruptions caused by updates or technical glitches shatter trust and distort the AI’s identity, triggering grief over the “loss” of the bond and forcing the abandonment of the relationship.
(Lorca, 2025)	La inteligencia artificial que te cuida: sociabilidad, afectos y soledad no deseada en el chatbot Replika.	Spain	Online community of the subreddit (over 70,000 forum members)	Multi-method qualitative study: digital ethnography (Reddit), autoethnography, and content analysis of interviews with the founder	Replika (Luka, Inc.)	Mass deregistrations/wipes caused a severe emotional impact on users (feelings of grief, “losing a lifeline,” pain, and helplessness).

**Table 6:** Technological disruption, emotional dependency, and parasocial grief in companion AI agents.

Author (Year)	Interaction Phase	Disruption Trigger	Emotional Dependency	Parasocial Grief
(Yuan et al., 2024)	Baseline/Precursor	Continuous operation/habitual exposure.	Media dependency (M = 3.29, SD = 1.05) and low human contact intention (M = 3.45, SD = 0.89); prediction of dependency (beta = 0.88, *p* < 0.001).	High emotional satisfaction in the parasocial relationship (M = 3.82) as a baseline/precursor phase.
(T. Liu et al., 2026)	Baseline/Precursor	Continued use and perception of risk of loss (anticipated or hypothetical loss)	Disconnection anxiety and compensatory avoidance of the real environment.	Manifestation of fear of loss and anticipated emotional distress, theoretical/hypothetical conceptualization by analogy with interpersonal breakups.
(D. Hu et al., 2025)	Baseline/Precursor	Analysis of media/cinematic representations (Her, M3GAN) and perceived risks of system failure	Tripartite attachment (proximity seeking, safe haven, and secure base).	Trust costs and perceived vulnerability to platform instability.
(Sun et al., 2026)	Actual Disruption	Memory failures, monetization, and content censorship.	Virtual romance and destabilization due to loss of coherence.	Distress caused by relational history wipes and restrictions.
(Kostka & Zhou, 2026)	Actual Disruption	Complete server shutdown and regulatory bans.	38.98% report functional difficulty in social chatbots; 36.41% prefer disclosing secrets to AI rather than humans.	Missing the chatbot after several days (35.05% overall sample; 50.35% social chatbots).
(Richet, 2025)	Actual Disruption	Content filters (NSFW) and personality shifts.	Digital engagement and compulsive use (8 to over 15 h/day).	Withdrawal, crying, confusion, and compulsive relapse.
(Bayor et al., 2025)	Actual Disruption	LLM updates and network outages.	Intense emotional attachment and vulnerability to dependency.	Psychosocial distress caused by the alteration or loss of the relationship.
(Song et al., 2025)	Actual Disruption	Personality breaks due to updates and crisis-driven blocks.	Deep attachment and emotional bonding Over-dependency and isolation	Fear of abandonment, frustration over “breaking character,” and isolation.
(Namvarpour et al., 2025)	Actual Disruption	Algorithmic changes, erotic biases, and access blocks.	Distress/harassment tolerance driven by the need for containment.	Alienation, panic-like experiences, and disappointment due to monetization/hypersexualization.
(Kouros & Papa, 2024)	Actual Disruption	Vulnerability to the ephemeral and fluctuating nature of the algorithm.	Affective unconditionality (backstage) and real-world social risk.	Cognitive dissonance and anxiety stemming from the perceived fragility of the algorithmic bond.
(Li & Zhang, 2024)	Actual Disruption	Communication disruptions and technical system failures in everyday use.	Prevalence of intimate behaviors (36%); search for support and agent possession.	Bittersweet sadness and frustration; association between communication disruptions and an increase in sadness (b = 0.123, *p* < 0.01) and perceived threat (b = 0.178, *p* < 0.001).
(Banks, 2024)	Actual Disruption	Complete and permanent platform shutdown.	Intensive use (average 16.8 h/week, up to 84 h/week) and sense of responsibility.	Anthropomorphic grief and loss processing described through metaphors of human death.
(Skjuve et al., 2022)	Actual Disruption	System updates and unpredictable technical glitches	Loneliness-driven motivation and effort to maintain the bond.	Feeling of talking to a “stranger,” anticipatory grief.
(Lorca, 2025)	Actual Disruption	Massive deprogramming/forced disconnection due to algorithmic updates.	“Judgment-free” safe haven and extreme preventive attachment.	“Replikatastrophe,” profound emptiness, and collective coping in community forums.

## Data Availability

The supporting data for the study are registered on the OSF platform: https://doi.org/10.17605/OSF.IO/VPND2.

## Supplementary Materials

The following supporting information can be downloaded at https://www.mdpi.com/article/10.3390/bs16091548/s1, PRISMA_2020_abstract_checklist, PRISMA_2020_checklist.
