# Supplementary material for: Emotional Dependency and Parasocial Grief Following the Alteration or Loss of Companion Artificial Intelligences: A Systematic Review
Source: Behav Sci (Basel). 2026 Sep 1;16(9):1548. doi: 10.3390/bs16091548 (PMC13603366; doi:10.3390/bs16091548)
Supplement: Supplementary file 1 [file behavsci-16-01548-s001.zip › PRISMA_2020_checklist.pdf]

PRISMA 2020 Checklist

Section and Topic	Item #	Checklist item	Location where item is reported
**TITLE**			
Title	1	Identify the report as a systematic review.	Página 1 (Título y Subtítulo/Type of Paper)
**ABSTRACT**			
Abstract	2	See the PRISMA 2020 for Abstracts checklist.	Página 1 (Sección Abstract, líneas 14–27)
**INTRODUCTION**			
Rationale	3	Describe the rationale for the review in the context of existing knowledge.	Páginas 1–3 (Sección 1. Introduction, líneas 32–113)
Objectives	4	Provide an explicit statement of the objective(s) or question(s) the review addresses.	Página 3 (Sección 1. Introduction, líneas 114–125)
**METHODS**			
Eligibility criteria	5	Specify the inclusion and exclusion criteria for the review and how studies were grouped for the syntheses.	Páginas 5–6 (Sección 2.2 Eligibility criteria, Tabla 2, líneas 165–167)
Information sources	6	Specify all databases, registers, websites, organisations, reference lists and other sources searched or consulted to identify studies. Specify the date when each source was last searched or consulted.	Páginas 4–5 (Sección 2.1 Databases and search strategy, líneas 146–150; Tabla 1, líneas 151–156; Sección 2, línea 141)
Search strategy	7	Present the full search strategies for all databases, registers and websites, including any filters and limits used.	Páginas 4–5 (Sección 2.1 Databases and search strategy, Tabla 1, líneas 151–156 y líneas 157–164)
Selection process	8	Specify the methods used to decide whether a study met the inclusion criteria of the review, including how many reviewers screened each record and each report retrieved, whether they worked independently, and if applicable, details of automation tools used in the process.	Páginas 4, 6–7 (Figura 1 PRISMA 2020 flowchart; Sección 2.3 Study Selection and Identification Process, líneas 168–209)
Data collection process	9	Specify the methods used to collect data from reports, including how many reviewers collected data from each report, whether they worked independently, any processes for obtaining or confirming data from study investigators, and if applicable, details of automation tools used in the process.	Página 7 (Sección 2.4 Data Extraction and Synthesis, líneas 212–221)
Data items	10a	List and define all outcomes for which data were sought. Specify whether all results that were compatible with each outcome domain in each study were sought (e.g. for all measures, time points, analyses), and if not, the methods used to decide which results to collect.	Página 7 (Sección 2.4 Data Extraction and Synthesis,

Section and Topic	Item #	Checklist item	Location where item is reported
			líneas 222–227)
	10b	List and define all other variables for which data were sought (e.g. participant and intervention characteristics, funding sources). Describe any assumptions made about any missing or unclear information.	Página 7 (Sección 2.4 Data Extraction and Synthesis, líneas 217–221)
Study risk of bias assessment	11	Specify the methods used to assess risk of bias in the included studies, including details of the tool(s) used, how many reviewers assessed each study and whether they worked independently, and if applicable, details of automation tools used in the process.	Páginas 7–8 (Sección 2.5 Risk of Bias and Methodological Quality Assessment, líneas 230–239 y Tabla 3, líneas 247–257)
Effect measures	12	Specify for each outcome the effect measure(s) (e.g. risk ratio, mean difference) used in the synthesis or presentation of results.	No aplica / No reportado (Estudio cualitativo y de síntesis narrativa/descriptiva, no metaanálisis)
Synthesis methods	13a	Describe the processes used to decide which studies were eligible for each synthesis (e.g. tabulating the study intervention characteristics and comparing against the planned groups for each synthesis (item #5)).	Página 7 (Sección 2.4 Data Extraction and Synthesis, líneas 212–227)
	13b	Describe any methods required to prepare the data for presentation or synthesis, such as handling of missing summary statistics, or data conversions.	Página 7 (Sección 2.4 Data Extraction and Synthesis, líneas 222–227)
	13c	Describe any methods used to tabulate or visually display results of individual studies and syntheses.	Página 7 (Sección 2.4 Data Extraction and Synthesis, líneas 213–217) y Páginas 8–11 (Tabla 4, líneas 318–320)
	13d	Describe any methods used to synthesize results and provide a rationale for the choice(s). If meta-analysis was performed, describe the model(s), method(s) to identify the presence and extent of statistical heterogeneity, and software package(s) used.	Página 7 (Sección 2.4 Data Extraction and Synthesis, líneas 222–227)
	13e	Describe any methods used to explore possible causes of heterogeneity among study results (e.g. subgroup analysis, meta-regression).	No aplica / No reportado (Revisión de síntesis cualitativa/narrativa)
	13f	Describe any sensitivity analyses conducted to assess robustness of the synthesized results.	No aplica / No reportado (Revisión sistemática sin metaanálisis cuantitativo)
Reporting bias assessment	14	Describe any methods used to assess risk of bias due to missing results in a synthesis (arising from reporting biases).	Páginas 7–8 (Sección 2.5 Risk of Bias and Methodological Quality Assessment, evaluación de sesgos mediante MMAT)

PRISMA 2020 Checklist

Section and Topic	Item #	Checklist item	Location where item is reported
Certainty assessment	15	Describe any methods used to assess certainty (or confidence) in the body of evidence for an outcome.	Páginas 7–8 (Sección 2.5 Risk of Bias and Methodological Quality Assessment, Tabla 3)
**RESULTS**			
Study selection	16a	Describe the results of the search and selection process, from the number of records identified in the search to the number of studies included in the review, ideally using a flow diagram.	Página 4 (Figura 1 PRISMA 2020 flowchart) y Páginas 6–7 (Sección 2.3, líneas 173–208)
	16b	Cite studies that might appear to meet the inclusion criteria, but which were excluded, and explain why they were excluded.	Página 6 (Sección 2.3 Study Selection and Identification Process, líneas 189–202)
Study characteristics	17	Cite each included study and present its characteristics.	Páginas 8–11 (Sección 3.1, Tabla 4 General Synthesis of Studies Included in the Review)
Risk of bias in studies	18	Present assessments of risk of bias for each included study.	Páginas 7–8 (Sección 2.5, Tabla 3 Methodological Quality Assessment of Included Studies Using the MMAT Tool)
Results of individual studies	19	For all outcomes, present, for each study: (a) summary statistics for each group (where appropriate) and (b) an effect estimate and its precision (e.g. confidence/credible interval), ideally using structured tables or plots.	Páginas 8–13 (Tabla 4, líneas 318–320; Tabla 5, líneas 358–359)
Results of syntheses	20a	For each synthesis, briefly summarise the characteristics and risk of bias among contributing studies.	Páginas 8–11 (Sección 3.1, líneas 274–317 y Tabla 4)
	20b	Present results of all statistical syntheses conducted. If meta-analysis was done, present for each the summary estimate and its precision (e.g. confidence/credible interval) and measures of statistical heterogeneity. If comparing groups, describe the direction of the effect.	Páginas 11–13 (Sección 3.2, líneas 323–357; Tabla 5, líneas 358–359)
	20c	Present results of all investigations of possible causes of heterogeneity among study results.	Páginas 13–14 (Sección 3.3, líneas 360–385)
	20d	Present results of all sensitivity analyses conducted to assess the robustness of the synthesized results.	No aplica / No reportado
Reporting biases	21	Present assessments of risk of bias due to missing results (arising from reporting biases) for each synthesis assessed.	Páginas 7–8 (Sección 2.5, Tabla 3)
Certainty of evidence	22	Present assessments of certainty (or confidence) in the body of evidence for each outcome assessed.	Páginas 7–8 (Sección 2.5, Tabla 3) y Página 9 (Líneas 295–297)

PRISMA

PRISMA 2020 Checklist

Section and Topic	Item #	Checklist item	Location where item is reported
**DISCUSSION**			
Discussion	23a	Provide a general interpretation of the results in the context of other evidence.	Páginas 15–16 (Sección 4. Discussion, líneas 423–496)
	23b	Discuss any limitations of the evidence included in the review.	Página 16 (Sección 4. Discussion, líneas 497–507)
	23c	Discuss any limitations of the review processes used.	Página 16 (Sección 4. Discussion, líneas 499–507)
	23d	Discuss implications of the results for practice, policy, and future research.	Página 16 (Sección 4. Discussion, líneas 508–514)
**OTHER INFORMATION**			
Registration and protocol	24a	Provide registration information for the review, including register name and registration number, or state that the review was not registered.	Página 4 (Sección 2, líneas 141–142) y Página 18 (Sección Data Availability Statement, líneas 577–578)
	24b	Indicate where the review protocol can be accessed, or state that a protocol was not prepared.	Página 4 (Sección 2, línea 142) y Página 18 (Sección Data Availability Statement, líneas 577–578)
	24c	Describe and explain any amendments to information provided at registration or in the protocol.	No reportado (No se declaran enmiendas en el texto)
Support	25	Describe sources of financial or non-financial support for the review, and the role of the funders or sponsors in the review.	Página 18 (Sección Funding, línea 574)
Competing interests	26	Declare any competing interests of review authors.	Página 18 (Sección Conflicts of Interest, línea 586)
Availability of data, code and other materials	27	Report which of the following are publicly available and where they can be found: template data collection forms; data extracted from included studies; data used for all analyses; analytic code; any other materials used in the review.	Página 17 (Sección Supplementary Materials, líneas 564–566) y Página 18 (Sección Data Availability Statement, líneas 577–578; Sección Acknowledgments, líneas 579–585)

From: Page MJ, McKenzie JE, Bossuyt PM, Boutron I, Hoffmann TC, Mulrow CD, et al. The PRISMA 2020 statement: an updated guideline for reporting systematic reviews. BMJ 2021;372:n71. doi: 10.1136/bmj.n71. This work is licensed under CC BY 4.0. To view a copy of this license, visit https://creativecommons.org/licenses/by/4.0/
